# First Evaluation of Ultrafast Ultrasound Coupled With Phrenic Stimulation for Noninvasive Diagnosis of Diaphragm Dysfunction

**DOI:** 10.1002/jcsm.70323

**Published:** 2026-06-17

**Authors:** Axel Nierding, Annelyse Nardi, Thomas Similowski, Christian Straus, Jean‐Luc Gennisson, Damien Bachasson

**Affiliations:** ^1^ Sorbonne Université, INSERM, UMRS1158 Neurophysiologie Respiratoire Expérimentale et Clinique Paris France; ^2^ Université Paris‐Saclay, Inserm, CNRS, CEA, Laboratoire d'Imagerie Biomédicale Multimodale (BioMaps) Orsay France; ^3^ Département R3S (Respiration, Réanimation, Réadaptation respiratoire, Sommeil), Service d'Explorations Fonctionnelles de la Respiration, de l'Exercice et de la Dyspnée AP‐HP, Groupe Hospitalier Universitaire APHP‐Sorbonne Université, Hôpital Pitié‐Salpêtrière Paris France; ^4^ Département R3S (Respiration, Réanimation, Réadaptation respiratoire, Sommeil) AP‐HP, Groupe Hospitalier Universitaire APHP‐Sorbonne Université, hôpital Pitié‐Salpêtrière Paris France; ^5^ Neuromuscular Investigation Center Institute of Myology Paris France

**Keywords:** diaphragm dysfunction diagnosis, noninvasive respiratory assessment, phrenic nerve stimulation, transdiaphragmatic twitch pressure, ultrafast ultrasound imaging

## Abstract

**Background:**

Diaphragm dysfunction is an important and often unrecognized cause of dyspnea. The current gold standard, transdiaphragmatic twitch pressure (Pdi,tw), requires oesophageal and gastric balloon catheters and is infrequently used in routine care. We evaluated whether ultrafast ultrasound descriptors of costal diaphragm during bilateral phrenic magnetic stimulation can provide a noninvasive alternative for assessing diaphragm contractility.

**Methods:**

Thirty patients (19 men and 11 women) referred for suspected diaphragm dysfunction (median age 57 [42–63] years) underwent bilateral anterolateral magnetic stimulation with simultaneous ultrafast ultrasound and oesophageal/gastric pressure recordings. Peak diaphragm tissue velocity, acceleration and jerk were extracted. Associations with Pdi,tw were assessed using ridge regression. Diagnostic performance for detecting abnormal Pdi,tw (< 20 cmH_2_O) was evaluated using Bayesian receiver operating characteristic (ROC) analysis, including posterior mean AUC and 95% credible intervals. Agreement between predicted and measured Pdi,tw was assessed using Lin's concordance correlation coefficient and Passing–Bablok regression.

**Results:**

Twenty four of 30 patients (80%) had abnormal Pdi,tw. Ultrafast ultrasound descriptors correlated with Pdi,tw (Spearman's *ρ*: velocity 0.77 [95% CI, 0.57–0.89], acceleration 0.70 [95% CI, 0.41–0.87], jerk 0.67 [95% CI, 0.43–0.85]; all *p* < 0.0001). The multivariable ridge model explained 66% of the variance in Pdi,tw and showed high agreement with measured values (Lin's concordance correlation coefficient = 0.87 [95% CI, 0.75–0.93]). Bayesian ROC analysis demonstrated strong discrimination of diaphragm dysfunction (AUC = 0.91; 95% credible interval [CrI], 0.76–0.98). Using the clinical threshold of 20 cmH_2_O, model‐predicted Pdi,tw yielded a sensitivity of 75% and specificity of 100%. The optimal velocity threshold for discriminating abnormal Pdi,tw was 10.25 mm·ms^−1^ (95% CrI, 6.12–18.58 mm·ms^−1^). The corresponding thresholds for acceleration and jerk were 408.6 mm·ms^−2^ (95% CrI, 122.6–952.4) and 3073 mm·ms^−3^ (95% CrI, 1038.8–11541.4), respectively.

**Conclusions:**

Ultrafast ultrasound coupled with magnetic phrenic stimulation provides a feasible, noninvasive, nonvolitional assessment of diaphragm contractility. Diaphragm motion descriptors reliably predicted Pdi,tw and enabled accurate identification of diaphragm dysfunction. These findings support further clinical evaluation and warrant larger multicentre validation studies.

## 1 Introduction

Diaphragm dysfunction is a frequently overlooked cause of dyspnea and should be systematically considered in the differential diagnosis of respiratory symptoms [1]. It encompasses a continuum from partial function loss to complete paralysis and may affect one or both hemidiaphragms. Aetiologies include direct trauma, surgery, neuromuscular diseases, phrenic nerve injury, disuse from mechanical ventilation and conditions associated with hyperinflation such as chronic obstructive pulmonary disease [1, 2, 3]. Despite its clinical impact, it remains underdiagnosed, partly due to the limitations of standard diagnostic tools [3, 4].

Diagnosis typically integrates clinical evaluation with imaging and physiological tests. Chest radiography is often the initial investigation, though its specificity is low [4]. Diaphragm elevation may also occur in nonneuromuscular conditions such as atelectasis, obesity or abdominal distension [3, 5]. Physiological assessments including sited/supine lung function testing, maximal inspiratory pressure and sniff nasal inspiratory pressure are useful for identifying a restrictive ventilatory defect and/or inspiratory weakness. Diaphragm ultrasound (US) has also emerged as a valuable, bedside imaging modality for assessing diaphragm structure and function [5]. However, these approaches are effort‐dependent and subject to interobserver variability but have shown good diagnostic performance in specialized settings [6, 7]. For definitive diagnosis, nonvolitional methods such as twitch transdiaphragmatic pressure elicited by phrenic stimulation (Pdi,tw) are often required. A Pdi,tw < 20 cmH_2_O is considered as the threshold for diaphragm dysfunction [6]. Despite its diagnostic specificity, Pdi,tw measurement is technically demanding, as it requires the placement of oesophageal and gastric catheters, careful control of lung volume and supramaximal stimulation of the phrenic nerves. Consequently, its clinical use remains restricted to specialized centres [7].

Ultrafast US of the costal diaphragm combined with magnetic phrenic stimulation has recently been proposed as a promising approach to bridge the gap between nonvolitional and noninvasive methods [8]. Ultrafast US, capable of frame rates of up to 20 kHz [9], enables the visualization of rapid muscle dynamics using radiofrequency speckle tracking and allows quantification of transient tissue velocities such as those recorded during skeletal muscle responses to artificial stimulation [10]. In healthy individuals, maximal diaphragm velocity (Vdi,max) has been shown to correlate with Pdi,tw and to be sensitive to the intensity of phrenic magnetic stimulation [8, 11]. However, its diagnostic performance in clinical populations has not been established.

Therefore, this study aimed to evaluate whether ultrafast US‐derived diaphragm motion descriptors during bilateral anterolateral magnetic stimulation could serve as surrogates for Pdi,tw in patients referred for suspected diaphragm dysfunction.

## 2 Methods

### 2.1 Ethical Approval

This study was approved by a regional ethics committee (*Comité de Protection des Personnes Sud Ouest et Outre Mer III*; IDRCB: 2021‐A01294‐37) and conducted in accordance with the Declaration of Helsinki. All patients provided written informed consent.

### 2.2 Participants

Patients with suspected diaphragmatic dysfunction were prospectively recruited from a specialized outpatient clinic dedicated to the evaluation of respiratory muscle function, where nonvolitional assessments, including Pdi,tw measurements and phrenic nerves conduction studies, are routinely performed. Suspicion of dysfunction was based on one or more of the following: unexplained dyspnea, postural decline in vital capacity, paradoxical abdominal motion, elevated hemidiaphragm on chest imaging or abnormal phrenic compound muscle action potentials (CMAPs). The presence of CMAP abnormalities was used together with other available clinical data (e.g., symptoms, spirometry and imaging) as supportive evidence for suspected diaphragm dysfunction.

Sample size was determined to detect a significant association between diaphragm US parameters and Pdi,tw with 80% power (*β* = 0.20) and *α* = 0.05. Previous studies demonstrated similar relationships with as few as 13 healthy subjects [8]. Assuming a large effect size (*ρ* ~ 0.7), power calculations indicated that at least 14 subjects would have been sufficient. However, 30 were recruited to improve statistical robustness and capture heterogeneity in a clinical population.

### 2.3 Pressure Measurements

Oesophageal (Pes) and gastric (Pga) pressures were measured using 8‐cm balloon‐catheters (Marquat Genie Biomedical, Boissy‐Saint‐Leger, France) inserted nasally after local anaesthesia. Balloons were filled with 1 mL (oesophageal) and 2 mL (gastric) of air and connected to differential pressure transducers (DP15, Northridge, CA, United States). Signals were sampled at 1 kHz (MEB 2300, Nihon Kohden, Tokyo, Japan) and recorded for offline analysis (Polaris, Nihon Kohden). Pdi was calculated as Pga–Pes. An abdominal belt was used to monitor abdominal expansion.

### 2.4 Phrenic Magnetic Stimulation

Bilateral anterior magnetic phrenic stimulation was performed using two 45‐mm figure‐of‐eight coils (90° handle) connected to synchronized Magstim 200 stimulators (Magstim, United Kingdom). Coils were positioned on the sternocleidomastoid muscles at the level of the cricoid to target the phrenic nerves. All participants tolerated stimulation without adverse events, and all sessions were completed as planned.

### 2.5 Ultrafast US Acquisitions

**FIGURE 1:**
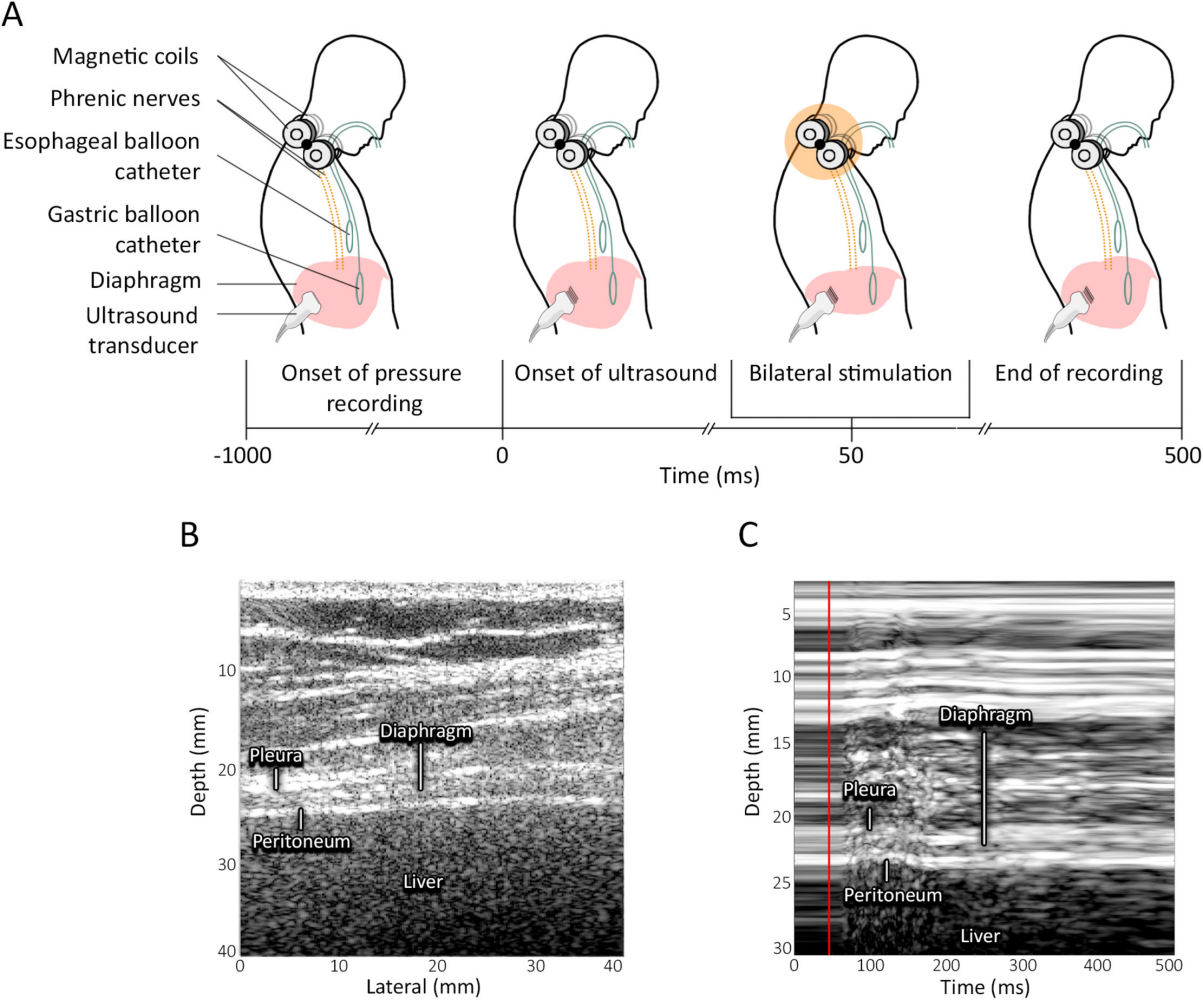
Experimental set‐up and procedure for pressure and ultrafast ultrasound (US) imaging of the diaphragm. (A) Each coil was positioned against the sternomastoid muscle, at the level of the cricoid cartilage. Pressure signal acquisition began 1000 ms before the onset of ultrasound imaging. Magnetic stimulation was delivered 50 ms after the start of the ultrafast ultrasound sequence, which lasted 500 ms. (B) Representative B‐mode image acquired using the custom sequence showing the pleura, diaphragm and peritoneum as distinct layers above the liver. (C) Time‐motion (M‐mode) representation extracted from the same sequence at a fixed lateral position, illustrating the temporal evolution of diaphragm motion before and after magnetic nerve stimulation. The vertical red line at 50 ms indicates the onset of magnetic stimulation.

Ultrafast US imaging was performed with a 6‐MHz linear transducer (SL 10‐2) and ultrafast system (Aixplorer V12, Supersonic Imagine, France). The right costal diaphragm was imaged at the zone of apposition, where the liver provides a stable, gas‐free acoustic window, yielding higher quality ultrafast data, with the probe placed along the mid‐axillary line between the 8th and 10th intercostal spaces (Figure 1). The diaphragm was visualized as a hypoechoic muscle layer bordered by hyperechoic pleural and peritoneal membranes. A custom ultrafast US sequence allowing 500‐ms acquisition was used as previously described [12]. US and pressure signals were synchronized by a trigger pulse from the US system to the acquisition unit. Stimulation was delivered 50 ms after the trigger to ensure time‐locked acquisition.

### 2.6 Experimental Protocol

Stimulations were delivered at 100% of stimulator output at previously determined optimal positions and at functional residual capacity, confirmed by Pes monitoring. Each participant received at least three stimulations, ≥ 1 min apart. Two to three recordings per patient were retained for analysis according to prespecified quality‐control criteria. Recordings were excluded if they showed (i) unstable prestimulation conditions (e.g., visible change in breathing pattern, drift in end‐expiratory Pes or patient movement), (ii) nonreproducible twitch responses (defined as > 20% variability in timing or amplitude compared with other trials in the same patient) [6] or (iii) technical artefacts (signal saturation or displacement of the US probe). Selection was performed by two investigators blinded to Pdi,tw outcomes, with discrepancies resolved by consensus. All 30 patients were retained in the analysis, with exclusions applied only at the stimulation level.

### 2.7 Data Analysis

All data were analysed offline using custom MATLAB scripts (The MathWorks, Natick, MA, United States). Pes, Pga and Pdi signals were filtered (30‐Hz cutoff), and twitch pressures (Pes,tw, Pga,tw and Pdi,tw) were calculated as the difference between baseline and peak values. A Pdi,tw < 20 cmH_2_O was considered abnormal according to current recommendations [7]. All acquisitions were performed by the same operator (AN), and analysis was blinded to clinical status and identity.

**FIGURE 2:**
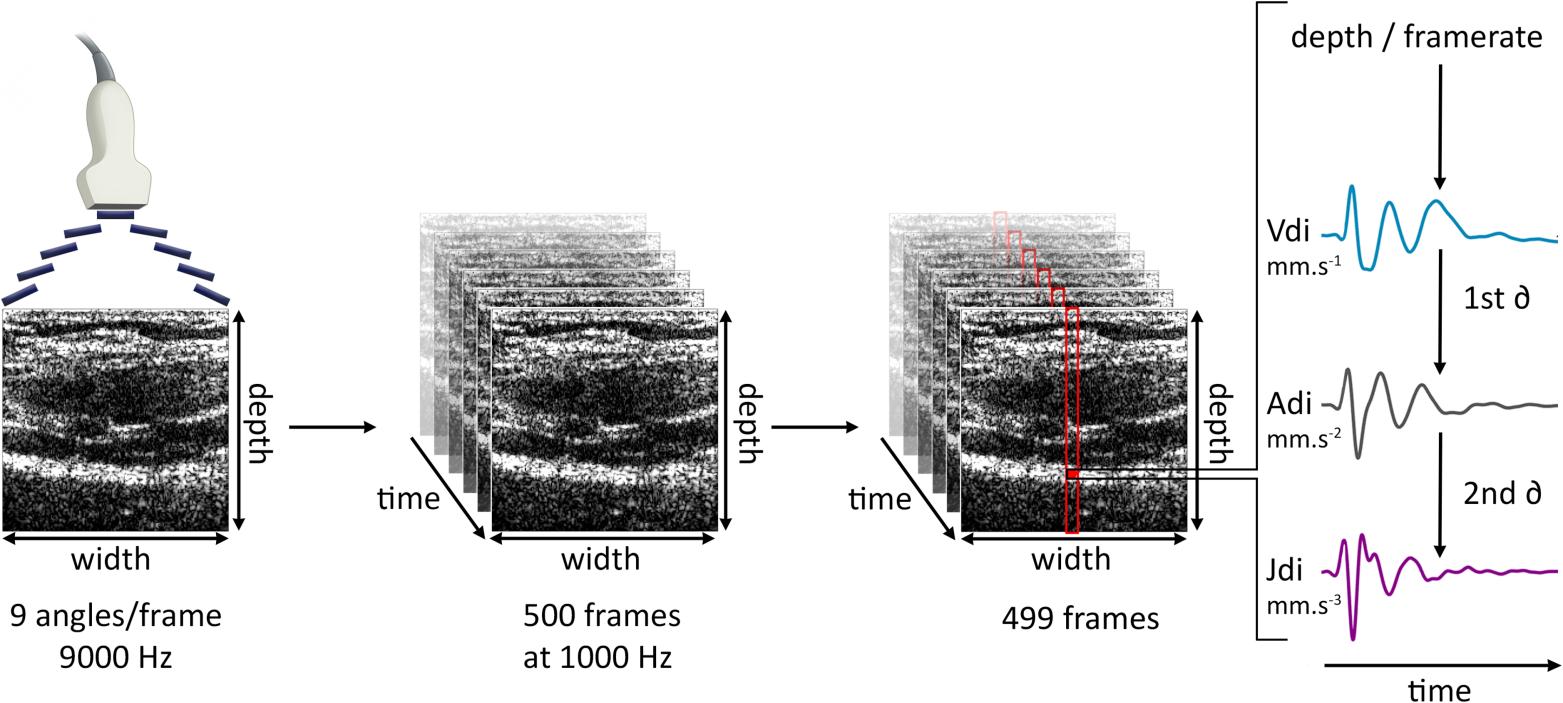
Workflow for extracting diaphragm velocity (Vdi), acceleration (Adi), and jerk (Jdi) from ultrafast ultrasound imaging. Ultrafast B‐mode images were acquired using a nine‐angle plane‐wave compounding sequence (−7° to +7°, pulse repetition frequency 9000 Hz) over a 500‐ms window, yielding 500 reconstructed frames at 1000 Hz. A central region of interest encompassing the diaphragm was tracked across consecutive frames using one‐dimensional axial cross‐correlation, producing a depth‐versus‐time displacement signal. Vdi was obtained as the first temporal derivative of displacement from the diaphragm region (red); Adi and Jdi were derived as the first and second temporal derivatives of Vdi, respectively.

Diaphragm motion was quantified using speckle‐tracking of ultrafast US images. Tissue velocity profiles were derived from axial pixel displacements, averaged across the central third of the image as previously described [8, 13] and shown in Figure 2. From these profiles, peak velocity (Vdi,max), acceleration (Adi,max) and jerk (Jdi,max) were extracted within 0–100 ms after stimulation. Peak velocity represents the maximal speed of diaphragm shortening, acceleration reflects the rate of pressure‐generating force development and jerk describes the rapidity of change in acceleration. Accurate assessment of acceleration and jerk requires ultrafast frame rates to resolve these brief twitch dynamics.

**FIGURE 3:**
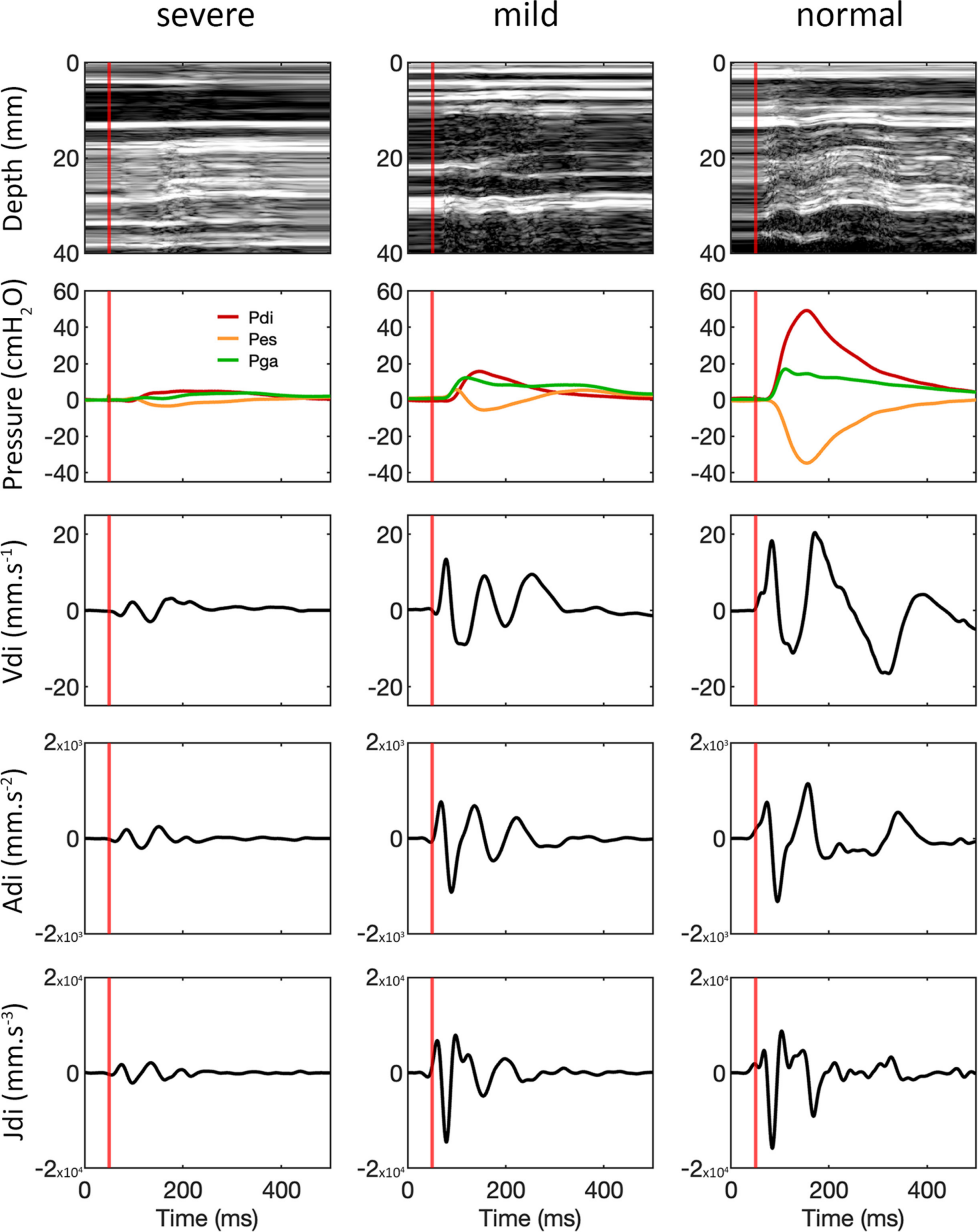
Representative ultrasound and pressure recordings in a patient with severe diaphragm dysfunction, a patient with mild diaphragm dysfunction and a patient with normal diaphragm function. Examples are shown from three individuals with severe (left), mild (middle) and normal (right) diaphragm function. Rows (top to bottom) display: time‐motion (M‐mode) image reconstructed from the central vertical line of each B‐mode frame; transdiaphragmatic (Pdi), oesophageal (Pes) and gastric (Pga) pressures; diaphragm tissue velocity (Vdi); diaphragm tissue acceleration (Adi); diaphragm tissue jerk (Jdi). The vertical red lines at 50 ms mark the onset of magnetic stimulation. All signals were recorded over a 500‐ms window.

Diaphragm contraction‐related abdominal motion was classified as ‘monophasic’ if followed by exponential decay or ‘biphasic’ if two peaks occurred. Representative recordings are shown in Figure 3. CMAPs were recorded using surface electrodes placed over the diaphragm during phrenic nerve stimulation. CMAPs reflect the summed electrical response of diaphragm motor units and are used to assess phrenic nerve conduction and neuromuscular transmission. Latency and peak‐to‐peak amplitude were measured from the averaged traces, from the stimulus artefact to the onset of the CMAP. CMAPs were considered abnormal when latency exceeded 8.5 ms and when amplitude was clearly reduced compared to previously observed values (i.e., < 0.3 mV) or the contralateral side (i.e., > 50% reduction) [7, 14, 15].

### 2.8 Statistical Analysis

Data normality was assessed using Q–Q plots, density plots and the Shapiro–Wilk test. Variables that were not normally distributed were analysed using Spearman's rank correlation coefficient (*ρ*). To model the relationship between ultrafast US metrics and Pdi,tw, ridge regression was applied to account for collinearity between predictors. The penalty parameter was optimized by cross‐validation, and predictive performance was assessed with a leave‐one‐subject‐out cross‐validation scheme, providing unbiased out‐of‐fold predictions for each patient. These predictions were used for all subsequent analyses [16, 17]. Predictive performance was compared with a mean‐only baseline model of Pdi,tw. A nonparametric bootstrap (1000 replications) provided 95% confidence intervals for the ridge model's root mean square error (RMSE). RMSE quantifies the average magnitude of the difference between predicted and measured Pdi,tw values and is expressed in the same physiological units (cmH_2_O), providing an interpretable measure of absolute prediction error.

Agreement between observed and predicted Pdi,tw values was quantified with Lin's concordance correlation coefficient (CCC). Passing‐Bablok regression [18] was applied to assess potential constant or proportional bias between predicted and observed values, with confidence intervals obtained by bootstrap resampling. This method is particularly appropriate here because it accounts for measurement error in both axes. In practice, this means that uncertainty is present not only in the US‐derived predictions (*x* axis) but also in the reference Pdi,tw measurements (*y* axis), which themselves are subject to biological variability, catheter positioning effects and stimulation‐related fluctuations. Ordinary least squares regression assumes that the *x* axis is measured without error, an assumption violated in physiological measurement studies. Passing‐Bablok treats both variables as imperfect measurements of the underlying true relationship, yielding slope and intercept estimates that remain valid even when errors occur in both dimensions.

Given the small size and imbalanced dataset (24 patients with diaphragm dysfunction and six without), conventional receiver operating analysis (ROC) could yield biased or unstable estimates. Therefore, a Bayesian ROC approach was used [19], which incorporates prior distribution to stabilize estimates in small, unbalanced samples. This framework models full posterior distributions of sensitivity, specificity and AUC, allowing uncertainty to be expressed through 95% credible intervals and providing a more robust assessment of diagnostic performance than classical ROC methods in this setting. In other words, the Bayesian ROC approach quantifies diagnostic performance as a probability distribution rather than a single point estimate, which is particularly important in small or imbalanced cohorts where frequentist ROC metrics can be unstable or overly optimistic. Posterior predictions were obtained from 2000 Markov chain Monte Carlo iterations across four chains. All analyses were conducted using R (R Core Team, 2024). A *p* value of < 0.05 was considered statistically significant. Continuous data are presented as median (Q1–Q3).

## 3 Results

### 3.1 Patients

Thirty patients (19 men and 11 women) were enrolled. Median age was 56.6 years [41.5–63], body mass index 28.3 kg m^−2^ [25.4–33.2], height 1.7 m [1.6–1.8] and weight 82 kg [73–96]. Vital capacity in the sitting position was 75% [62–86] of the predicted value [20] and 66% [56–79] in the supine position. Median postural decline of vital capacity in the supine position was 8% [4–23]; four patients had > 15%, and five had > 30% decline. Maximal inspiratory and sniff inspiratory pressure were 80.5% [68.0–99.2] and 51% [29–59] of the predicted values [21, 22].

Based on CMAP latency, six patients had bilateral diaphragm dysfunction, eight had isolated left hemidiaphragm dysfunction and six had isolated right hemidiaphragm dysfunction. Chest radiography showed left‐sided elevation in eight patients, right‐sided in six and suspected bilateral involvement in six. Clinical characteristics are summarized in Table 1.

**TABLE 1:** Study population characteristics.

Variable	Total population (*N* = 30)
Corticotherapy	7 (23.3%)
Respiratory support	12 (40%)
Dyspnea (Sadoul)	1.5 [1–2.8]
Orthopnea	10 (33.3%)
Dyspnea when lying on one side	7 (23.3%)
Dyspnea on bending forward	16 (53.3%)
Epworth score (/24)	9 [5–14]
> 11	14 (46.7%)
Chest X‐ray asymmetry	15 (50%)
Position of the right hemidiaphragm (intercostal space)	7th [5–7]
Position of the left hemidiaphragm (intercostal space)	7th [5–7]
CV sitting (L)	3.1 [2–3.7]
CV supine (L)	2.6 [1.8–3.2]
Postural decline (%)	8% [4–18]
MIP (cmH_2_O)	83 [61.8–99]
SNIP (cmH_2_O)	46 [26–65]
Pes before stimulation (cmH_2_O)	0.01 [−0.24–0.24]
Pdi,tw (cmH_2_O)	12.8 [7.82–20.3]
< 20 cmH_2_O	24 (80%)
Vdi,max (mm·s^−1^)	7.0 [4.72–10.8]
Adi,max (mm·s^−2^)	300 [194–571]
Jdi,max (mm·s^−3^)	2946 [1897–5975]
Biphasic abdominal expansion	10 (33.3%)
Right CMAP latency (ms)	7.9 [7.1–9.5]
> 8.5 (ms)	6 (20%)
Left CMAP latency (ms)	8.2 [7.2–9.4]
> 8.5 (ms)	8 (26.7%)
Right CMAP amplitude (μV)	436 [287–656]
Left CMAP amplitude (μV)	461 [255–815]

*Note:* Results are shown as median (Q1–Q3) or *n* (%).

Abbreviations: Adi,max, maximal diaphragm tissue acceleration; CMAP, compound motor action potential; CV, vital capacity; Jdi,max, maximal diaphragm tissue jerk; MIP, maximal inspiratory pressure; Pdi,tw, twitch transdiaphragmatic pressure; SNIP, sniff inspiratory pressure; Vdi,max, maximal diaphragm tissue velocity.

### 3.2 Relationship Between Transdiaphragmatic Pressure and Ultrafast US‐Derived Variables (Patient‐Averaged Analysis)

**FIGURE 4:**
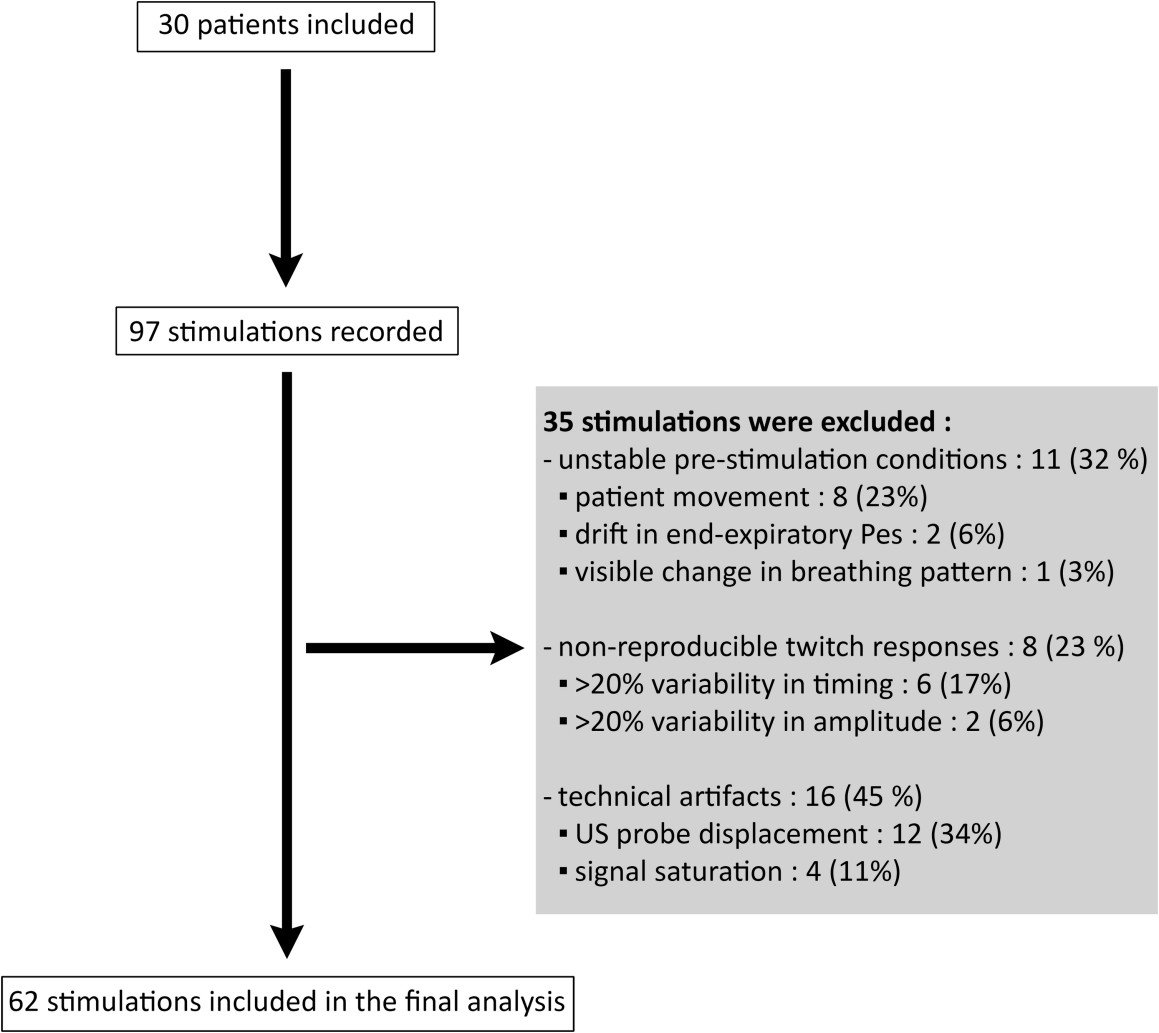
Flowchart of patient inclusion and stimulation selection.

Overall, 97 stimulations were recorded in 30 patients; 35 stimulations (36%) were excluded because of unstable prestimulation conditions (*n* = 11), nonreproducible twitch responses (*n* = 8) or technical artefacts (*n* = 16), so that 62 stimulations were retained for analysis (Figure 4).

**FIGURE 5:**
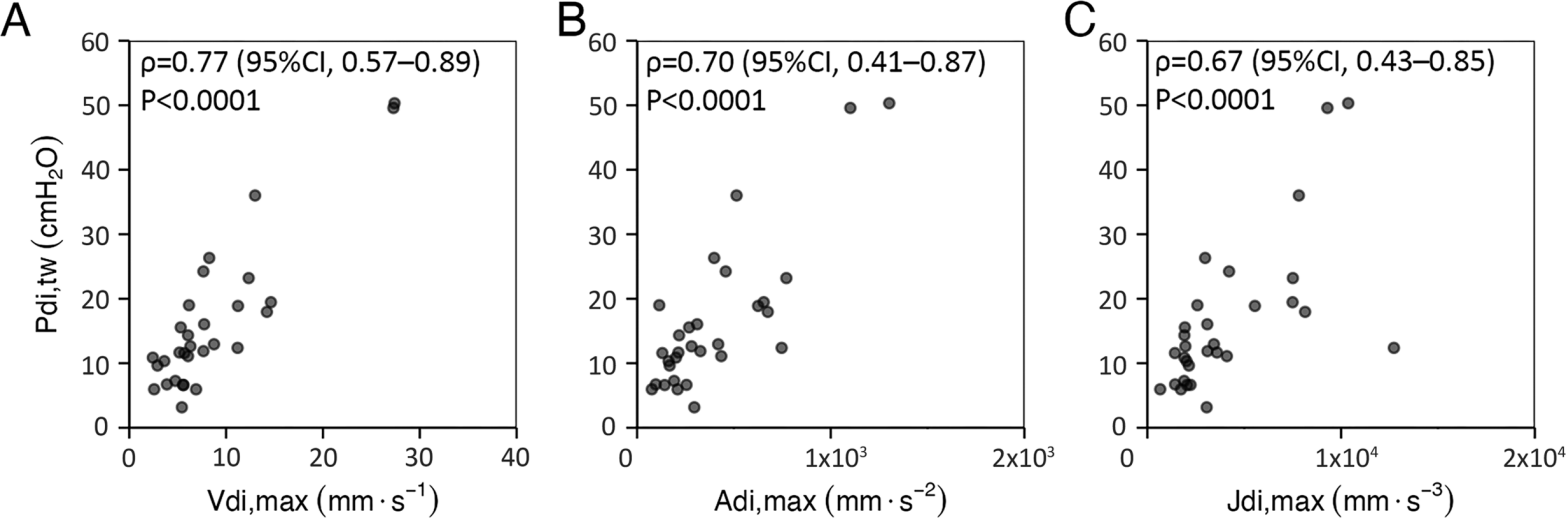
Relationship between ultrafast ultrasound‐derived variables and twitch transdiaphragmatic pressure (patient‐averaged data). Scatterplots showing the relationship between twitch transdiaphragmatic pressure (Pdi,tw) and (A) maximal diaphragm tissue velocity (Vdi,max), (B) maximal acceleration (Adi,max) and (C) jerk (Jdi,max) across all individual stimulations (*n* = 62). Each point represents one participant (*N* = 30, based on averaged values from two to three accepted stimulations per individual).

Patient‐averaged values of US variables were strongly correlated with Pdi,tw: Vdi,max (*ρ* = 0.77 [95% CI, 0.57–0.89]), Adi,max (*ρ* = 0.70 [95% CI, 0.41–0.87]) and Jdi,max (*ρ* = 0.67 [95% CI, 0.43, 0.85]), all *p* < 0.0001 (Figure 5).

In predictive analyses, Vdi,max alone explained 69% of the variance in Pdi,tw (*R* ^2^ = 0.69; 95% CI, 0.26–0.90) with an RMSE of 5.52 cmH_2_O (95% CI, 4.09–6.86), outperforming both Adi,max (*R* ^2^ = 0.55; 95% CI, 0.10–0.84; RMSE = 6.83 cmH_2_O) and Jdi,max (*R* ^2^ = 0.26; 95% CI, −0.21–0.79; RMSE = 8.90 cmH_2_O). The full ridge regression model including Vdi,max, Adi,max and Jdi,max explained 66% of the variance (*R* ^2^ = 0.66; 95% CI, 0.20–0.88) with an RMSE of 5.84 cmH_2_O (95% CI, 4.24–7.48), lower than the baseline mean‐only model (RMSE = 11.22 cmH_2_O; 95% CI, 5.93–15.29), with the following expression:

(1) $$\begin{array}{c} \mathrm{Pdi},\mathrm{tw}=3.10+1.26\times \mathrm{Vdi},\max+9.81\times \;10^{‐3}\times \mathrm{Adi},\max\\ \;-3.426\times 10^{‐4}\times \mathrm{Jdi},\max \end{array}$$

**FIGURE 6:**
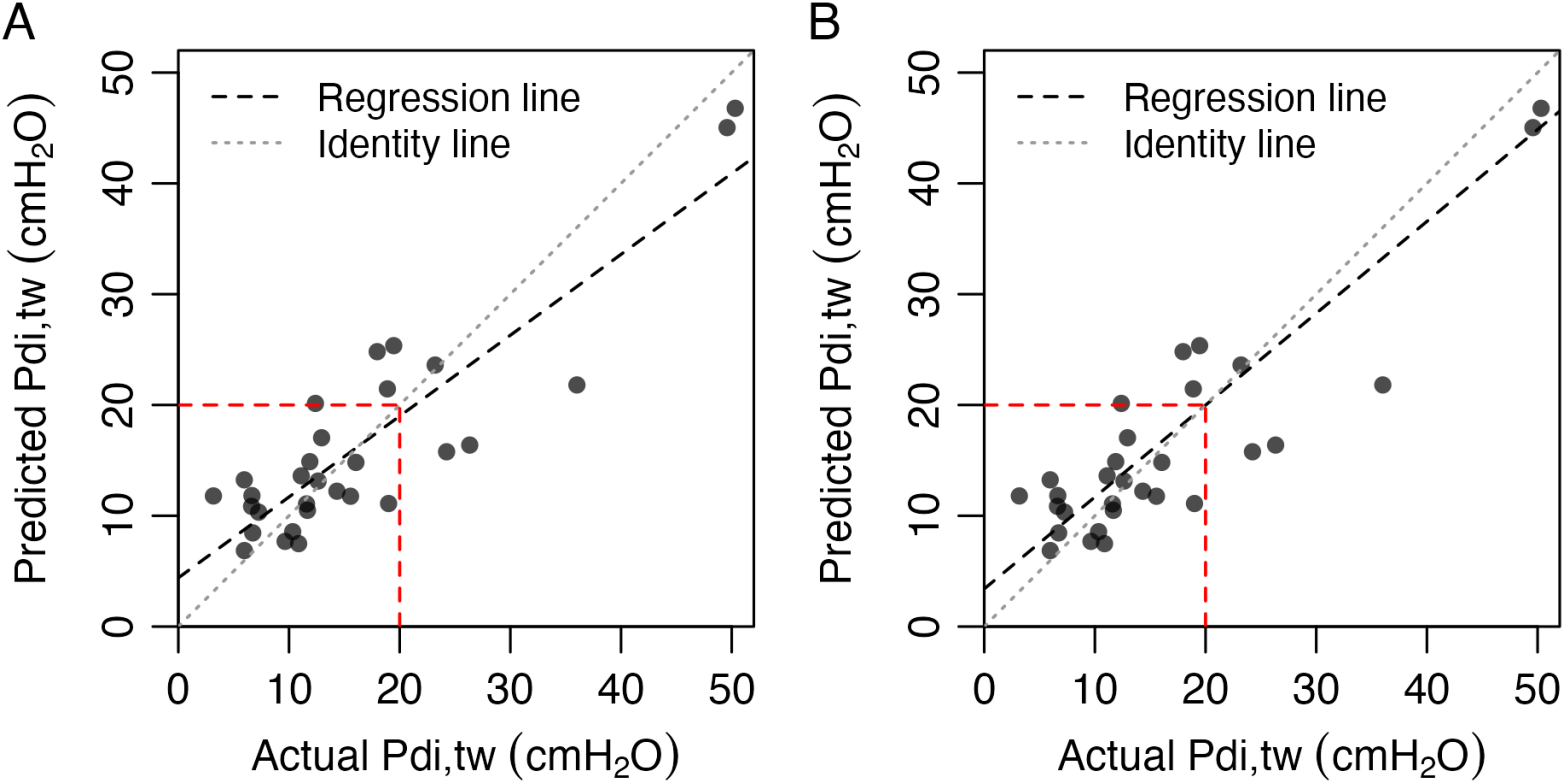
Calibration and agreement between actual and model‐predicted transdiaphragmatic pressure on patient‐averaged data (*n* = 30). (A) Ridge regression model of actual versus predicted Pdi,tw with the OLS calibration line. (B) Passing‐Bablok method‐comparison fit of predicted on actual. Both panels refer to the same multivariable ridge model that incorporates Vdi,max, Adi,max and Jdi,max. Red dashed segments mark the clinical cut‐off at 20 cmH_2_O. Predictions are leave‐one‐subject‐out to limit optimism. Pdi,tw, twitch transdiaphragmatic pressure.

Vdi,max and Adi,max were positively associated with Pdi,tw, whereas Jdi,max retained a negative association. Only Vdi,max remained a significant independent predictor in multivariable analyses. Agreement between observed and predicted values was high (CCC = 0.87; 95% CI, 0.75–0.93). Passing–Bablok regression yielded a slope of 0.81 (95% CI, 0.44–1.14) with an intercept of 3.12 (95% CI, −1.11 to 7.24) [15]. Figure 6 illustrates the relationship between measured and model‐predicted transdiaphragmatic pressure.

### 3.3 Diagnostic Performance of Ultrafast US‐Derived Variables

**FIGURE 7:**
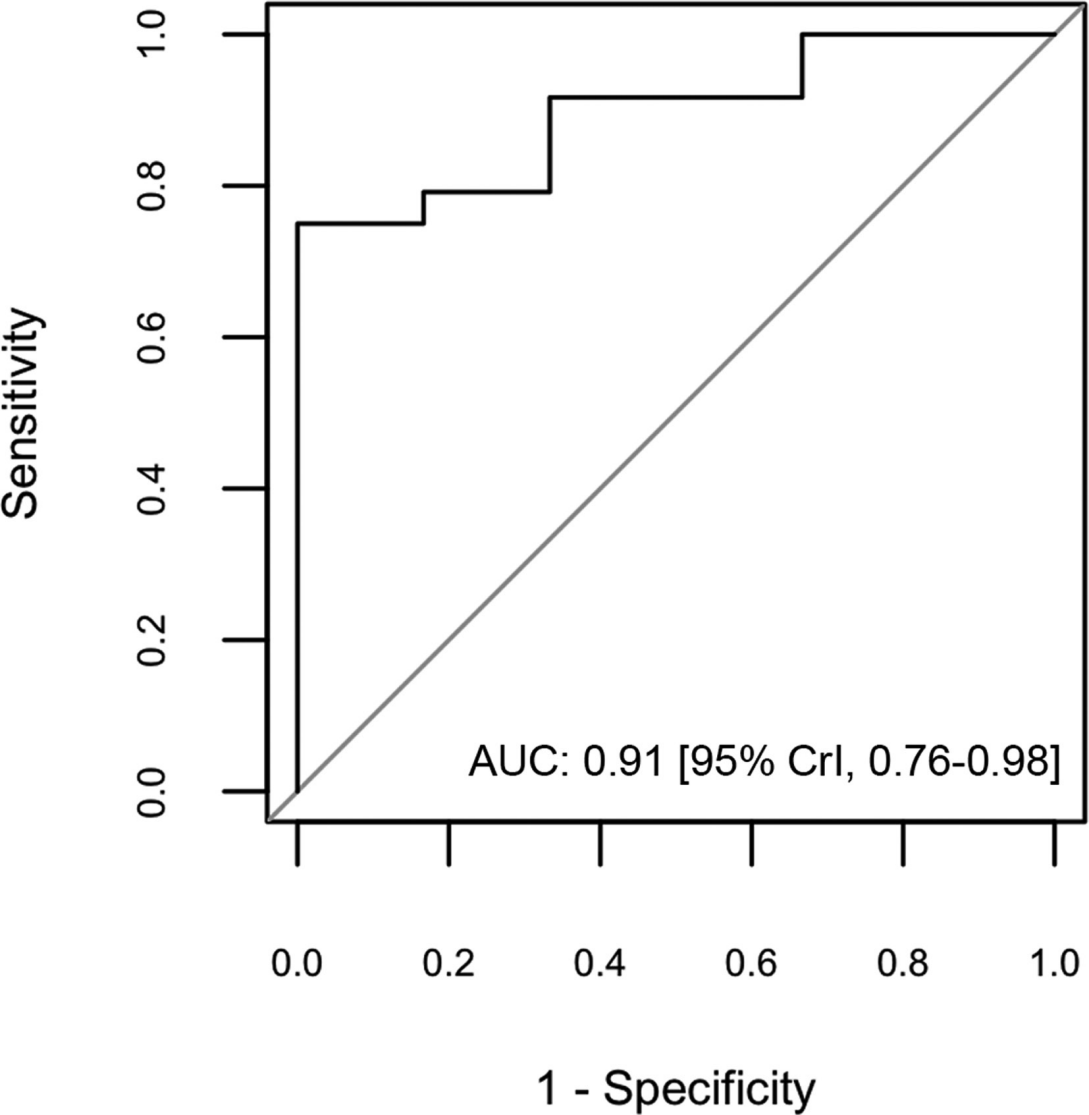
Bayesian receiver operating characteristic (ROC) curves for the multivariable ridge model based on Vdi,max, Adi,max and Jdi,max. ROC curves generated from Bayesian multiple logistic regression models using combined‐variable model: maximal diaphragm tissue velocity, acceleration and jerk. The area under the curve (AUC) model's ability to discriminate between patients with preserved versus abnormal twitch transdiaphragmatic pressure.

The Bayesian ROC curve for the multivariable model (Vdi,max, Adi,max and Jdi,max) yielded an AUC of 0.91 (95% CrI, 0.76–0.98) (Figure 7). At the optimal cut‐point defined by the Youden index (predicted Pdi,tw < 20 cmH_2_O), the model achieved a sensitivity of 0.75 (95% CrI, 0.67–0.79) and a specificity of 1.00 (95% CrI, 1.00–1.00), corresponding to a Youden index of 0.75 (95% CrI, 0.67–0.79). The Bayesian ROC curve for the univariate model (Vdi,max, Adi,max and Jdi,max) yielded an AUC of 0.90 (95% CrI, 0.75–0.98), 0.91 (95% CrI, 0.77–0.98) and 0.87 (95% CrI, 0.70–0.97), respectively. The optimal Vdi,max threshold for discriminating abnormal Pdi,tw was 10.25 mm·ms^−1^ (95% CrI, 6.12–18.58 mm·ms^−1^). The corresponding thresholds for Adi,max and Jdi,max were 408.6 mm·ms^−2^ (95% CrI, 122.6–952.4) and 03073.2 mm·ms^−3^ (95% CrI, 1038.9–0.11541.4), respectively.

## 4 Discussion

To our knowledge, this is the first clinical study to harness ultrafast US imaging during phrenic nerve stimulation in patients with suspected diaphragm dysfunction in routine care. The ultrafast approach is essential because phrenic nerve stimulation evokes a very brief twitch contraction (~200 ms). Conventional US, limited to tens of frames per second, cannot capture these rapid dynamics with sufficient temporal fidelity, whereas ultrafast imaging resolves the transient changes in velocity, acceleration and jerk. Our primary finding is that US‐derived motion variables were significantly associated with Pdi,tw, with velocity showing the strongest and most clinically interpretable relationship. Diagnostic accuracy for identifying abnormal Pdi,tw responses was high, supporting the feasibility of this method as a noninvasive, nonvolitional alternative to catheter‐based measurements.

Velocity showed the strongest correlation with Pdi,tw (Figure 5A), in line with prior observations in healthy participants and reinforcing its value as a proxy for overall diaphragm pressure‐generating capacity [8]. This is consistent with previous ultrafast US studies in limb muscles, such as the *biceps brachii* and quadriceps, where tissue velocity reliably reflects contractile output [10, 23, 24]. Additionally, Illidi and Romer [25] reported that subcostal excursion velocity, measured with conventional US during phrenic stimulation, correlated with Pdi,tw in healthy participants [26]. Acceleration was also significantly associated with Pdi,tw (Figure 5B) consistent with earlier observations that a faster rate of force development is linked to higher peak twitch responses [27]. These converging findings support the relevance of motion‐based variables over geometric metrics (e.g., thickening fraction), which have shown weaker associations with diaphragm contractile function in both animal and human studies [26, 28, 29]. Combining subcostal and intercostal imaging may further improve the characterization of diaphragmatic twitch contraction.

Whereas velocity, acceleration or jerk alone explained a substantial portion of Pdi,tw variance, residual variability likely reflects physiological and technical factors. Magnetic stimulation can activate other inspiratory muscles such as sternocleidomastoids and scalenes, generating intrathoracic pressure independently of diaphragm contraction [30]. Additionally, asymmetrical diaphragm involvement (e.g., unilateral phrenic paralysis) introduces complex motion patterns such as paradoxical elevation of the paralysed hemidiaphragm, driven by abdominal pressure generated by the descent of the contralateral healthy side. This phenomenon, well described in animal models [31], reduces Pdi independently of muscle contractility, which also explains the functional improvement observed after surgical diaphragm plication despite unchanged intrinsic contractility [32]. Such asymmetrical and nonuniform dynamics cannot be fully captured with 2D imaging. Minor deviations in transducer position or angle may also affect signal quality and speckle‐tracking results.

The multivariable ridge regression model outperformed the baseline model (mean‐only), reducing unexplained variance to approximately 34%. Velocity and acceleration showed positive associations with Pdi,tw, with velocity emerging as the sole significant independent predictor. Taken together, these considerations indicate that Vdi,max should be viewed as the primary, clinically actionable parameter at present, whereas the multivariable model serves to explore the incremental diagnostic information embedded in the full displacement‐velocity‐acceleration‐jerk profile. As larger datasets become available, these higher order indices may help refine phenotyping of diaphragm dysfunction and support more sophisticated modelling strategies, but such applications remain preliminary in the current study. From a clinical standpoint, an RMSE of approximately 5–6 cmH_2_O indicates that US‐based predictions deviate from measured Pdi,tw by about this magnitude on average. This level of error is substantially smaller than the pressure thresholds typically used to define clinically meaningful diaphragm dysfunction (i.e., Pdi,tw < 20 cmH_2_O). Accordingly, the model's prediction accuracy falls within a range compatible with clinical interpretation, although individual‐level variability remains and external validation will be required to confirm generalizability. Interestingly, Jdi,max was inversely associated with Pdi,tw in the multivariable model, despite a positive correlation in univariate analysis (Figure 5C). This likely reflects a statistical suppressor effect, whereby the inclusion of jerk improves model performance by accounting for variance not directly related to pressure output [33]. In muscle mechanics, a high positive jerk marks abrupt onset or rapid modulation of motion, whereas oscillatory or erratic jerk can indicate irregular or desynchronized recruitment of motor units. Physiologically, elevated jerk can reflect asynchrony, brief twitch‐like transients or unbalanced activation (e.g., accessory muscles vs. diaphragm) [34, 35]. Notably, the association between Jdi,max and Pdi,tw was absent in patients exhibiting biphasic diaphragm contraction‐related abdominal motion as compared to that observed in patients with monophasic diaphragm contraction‐related abdominal motion (*ρ* = 0.24, *p* = 0.29 vs. *ρ* = 0.70, *p* < 0.0001). This may indicate a loss of temporal alignment between motion signals and true contractile force in cases of unbalanced activation. Targeted experimental studies are needed to clarify whether jerk primarily indexes onset abruptness, coordination quality or both.

Averaging across stimulations reduced intrasubject variability, resulting in more stable and generalizable predictions of diaphragm contractile behaviour. When Vdi,max, Adi,max and Jdi,max are set to zero, the model still predicts a small residual Pdi,tw (~3–4 cmH_2_O). This residual pressure most likely arises from measurement artefacts, such as minimal probe displacement or minor thoracoabdominal movements occurring during magnetic stimulation. The persistence of this residual output is also a known feature of regularized regression models, which tend to avoid predicting exact zero values due to penalty constraints.

Although the averaged model improved overall accuracy, prediction errors persist at the individual level, likely reflecting partial recruitment, asymmetry and/or measurement errors. However, the Passing‐Bablok regression indicated acceptable agreement between US‐derived variables and the gold standard, with a slope close to unity and an intercept near zero, supporting the interchangeability of methods. Collectively, these findings underscore the promise of composite, motion‐derived US models for noninvasive assessment of diaphragm contractility.

To evaluate classification performance, we used a Bayesian ROC framework, which provides robust estimates with small or imbalanced datasets [19, 36]. The multivariable model achieved an AUC qualified as ‘good’ [37], suggesting that this method may be particularly valuable for confirming diaphragm dysfunction in patients with ambiguous clinical or volitional test results. Nonetheless, further external validation on independent cohorts is required to confirm reproducibility and clinical applicability across different populations and settings. In addition to the multivariable analysis, we explored univariable ROC models to provide preliminary thresholds for each mechanical descriptor. These cut‐offs should be regarded as exploratory, as they were derived from a single‐centre cohort with an imbalanced distribution of diaphragm dysfunction. Their stability is therefore limited, and they will require validation and potential recalibration in larger and more heterogeneous populations before being considered for clinical decision‐making.

Several limitations should be acknowledged. Accordingly, US‐derived motion indices should be interpreted as reflecting the diaphragm's net pressure‐generating capacity under standardized conditions, rather than intrinsic muscle strength, as they are influenced by the same neuromechanical and loading factors that determine Pdi,tw. The modest sample size may limit generalizability, and the absence of an external validation cohort restricts interpretations regarding the robustness of predictions. The predictive model did not include demographic or anthropometric variables as covariates. This proof‐of‐concept study was designed to characterize the relationship between ultrafast US metrics and simultaneously measured Pdi,tw, rather than to derive a fully adjusted prediction equation. Larger cohorts will be needed to determine whether factors such as sex, age, body habitus or underlying disease modify these relationships and to develop population‐specific reference values if appropriate. Our Bayesian ROC approach and internal bootstrap help mitigate overfitting, but larger, balanced cohorts are needed. Ultrafast US imaging was limited to the right hemidiaphragm, leaving potential asymmetries unexplored. Future studies should aim to incorporate bilateral measurement to improve side‐by‐side evaluation and understanding of diaphragm mechanics. Correlations between Vdi,max and Pdi,tw were comparable in right‐ and left‐sided paralysis (*ρ* = 0.71 vs. 0.67), though the small difference may reflect anatomical or mechanical asymmetries. For instance, hepatic support on the right side may limit paradoxical motion, making mechanical correlations potentially more robust in right‐sided dysfunction. Importantly, prior work by Verin et al. [38] demonstrated that oesophageal pressure responses to unilateral stimulation of the healthy side significantly correlate with those from bilateral stimulation in unilateral paralysis, supporting the relevance of side‐specific measures to global pressure generation. As a result, right‐sided kinematic variables may overestimate global pressure output in left‐sided dysfunction, weakening the observed correlation. However, our limited subgroup sizes prevent definitive conclusions. Bilateral ultrafast imaging also remains to be explored to address this potential limitation. Conventional diaphragm US indices such as excursion and thickening fraction [39, 40] during volitional inspiratory manoeuvres were not acquired because the protocol was restricted to twitch‐based assessment against the gold‐standard reference Pdi,tw. Although appropriate for validating ultrafast US twitch imaging, this design prevents a direct, within‐patients comparison with conventional US imaging during volitional manoeuvres. Future studies should therefore combine both approaches to quantify the added diagnostic value of ultrafast US during phrenic stimulation. All ultrafast acquisitions were performed by a single operator, and the protocol was not designed for formal reproducibility testing, so intraobserver and interobserver reliability could not be assessed. This limits external reproducibility and future studies should include multioperator imaging to quantify intraobserver and interobserver variability. Although stimulation was generally well tolerated, mild discomfort is expected and may limit routine use compared with fully passive US imaging. Although this investigation focused on ambulatory patients, the same nonvolitional ultrafast protocol could be applied in nonintubated critically ill patients and in pulmonary or neuromuscular disorders, in whom twitch Pdi is rarely measured in routine care. In invasively ventilated patients, twitch airway pressure already provides a simple noninvasive surrogate of Pdi,tw, so the present US approach should be viewed as complementary rather than a replacement in that setting. The strong association with Pdi,tw suggests this method may enable longitudinal monitoring of diaphragm contractile function. Its potential for longitudinal assessment is further supported by prior findings showing that Vdi,max is sensitive to stimulation intensity [8] and by Illidi and Romer's [25] demonstration that excursion velocity responds to fatigue in healthy individuals. Finally, technical improvements such as probe stabilization and automated analysis will be important for clinical translation.

In conclusion, ultrafast US during phrenic stimulation provides a noninvasive and effort‐independent method to assess diaphragm contractility. The present study shows encouraging agreement with reference techniques and a high ability to identify diaphragm dysfunction, although these findings remain exploratory. Larger and independent cohorts are required to confirm performance, to define robust reference values and to determine how this approach can best complement current invasive standards or serve as a noninvasive surrogate for them in appropriate clinical settings.

## Funding

This study was supported by the Foundation EDF, the AFM‐Telethon and the French National Research Agency (ANR‐21‐ce‐19‐0052).

## Conflicts of Interest

T.S., J.‐L.G. and D.B. are listed as inventors on a patent that covers aspects of the approach used in this study. All other authors declare no conflicts of interest.
